# Supplementary material for: The Lack of STING Impairs the MHC-I Dependent Antigen Presentation and JAK/STAT Signaling in Murine Macrophages
Source: Int J Mol Sci. 2022 Nov 17;23(22):14232. doi: 10.3390/ijms232214232 (PMC9697192; doi:10.3390/ijms232214232)
Supplement: Supplementary file 1 [file ijms-23-14232-s001.zip › ijms-1866277-supplementary.pdf]

The lack of STING impairs the MHC-I dependent antigen presentation and JAK/STAT signaling in murine macrophages

Carmen Caiazza [1], Teresa Brusco [1], Federica D'Alessio [1], Massimo D'Agostino [1], Angelica Avagliano [2], Alessandro Arcucci [2], Concetta Ambrosino [3,4,5], Giuseppe Fiume [6,*,†] and Massimo Mallardo [1,*,†]

[1] Department of Molecular Medicine and Medical Biotechnology, University of Naples "Federico II", Via S. Pansini 5, 80131 Naples, Italy

[2] Department of Public Health, University of Naples "Federico II", Via S. Pansini 5, 80131 Naples, Italy

[3] Department of Science and Technology, University of Sannio, Via De Sanctis, 82100 Benevento, Italy

[4] IRGS, Biogem-Scarl, Via Camporeale, Ariano Irpino, 83031 Avellino, Italy

[5] IEOS-CNR, Via Pansini 6, 80131 Naples, Italy

[6] Department of Experimental and Clinical Medicine, University of Catanzaro "Magna Graecia", 88100 Catanzaro, Italy

* Correspondence: fiume@unicz.it (G.F.); massimo.mallardo@unina.it (M.M.)

† These authors share last authorship.

Supplementary Figures

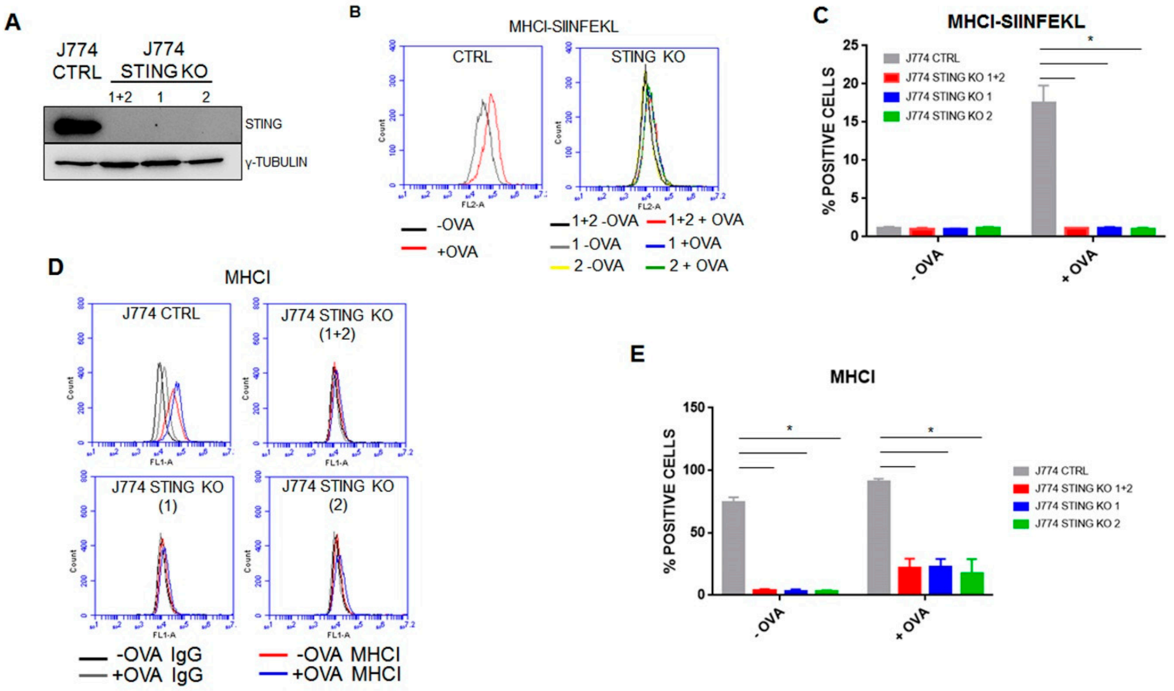

Figure S1. Efficiency of single RNA guides in silencing STING expression and in reducing the amount of MHC-I and MHC-I-SIINFEKL complex, upon OVA treatment. (A) J774 cells ($5x10^6$) were infected with scrambled vector, gRNA1, gRNA2 or gRNA1+2. Whole cell extracts (30 µg) were analyzed by western blot using the indicated antibodies. (B) J774 CTRL, STING KO 1, STING KO 2 and STING KO 1+2 ($1x10^6$) were treated with 500 µg/ml of OVA for 24h and were stained with SIINFEKL/H-2Kb-PE and IgG-PE, as control. Each plot represents 10000 events of a representative experiment. (C) Percentage of SIINFEKL/H-2Kb positive population in untreated or OVA treated cells. Values (mean ± SE, n = 3) are shown. The asterisks indicate a statistically significant difference compared to untreated control, according to Student's t-test ($p<0.01$). (D) J774 CTRL, STING KO 1, 2 and 1+2 cells ($1x10^6$) were treated with 500 µg/ml of OVA for 24h and stained with MHC-I-FITC and IgG-FITC as control. Each plot represents 10000 events of a representative experiment. (E) Percentage of MHCI positive population in untreated or OVA treated cells. Values

(mean ± SE, n = 3) are shown. The asterisks indicate a statistically significant difference compared to untreated control, according to Student's t-test ($p<0.01$).

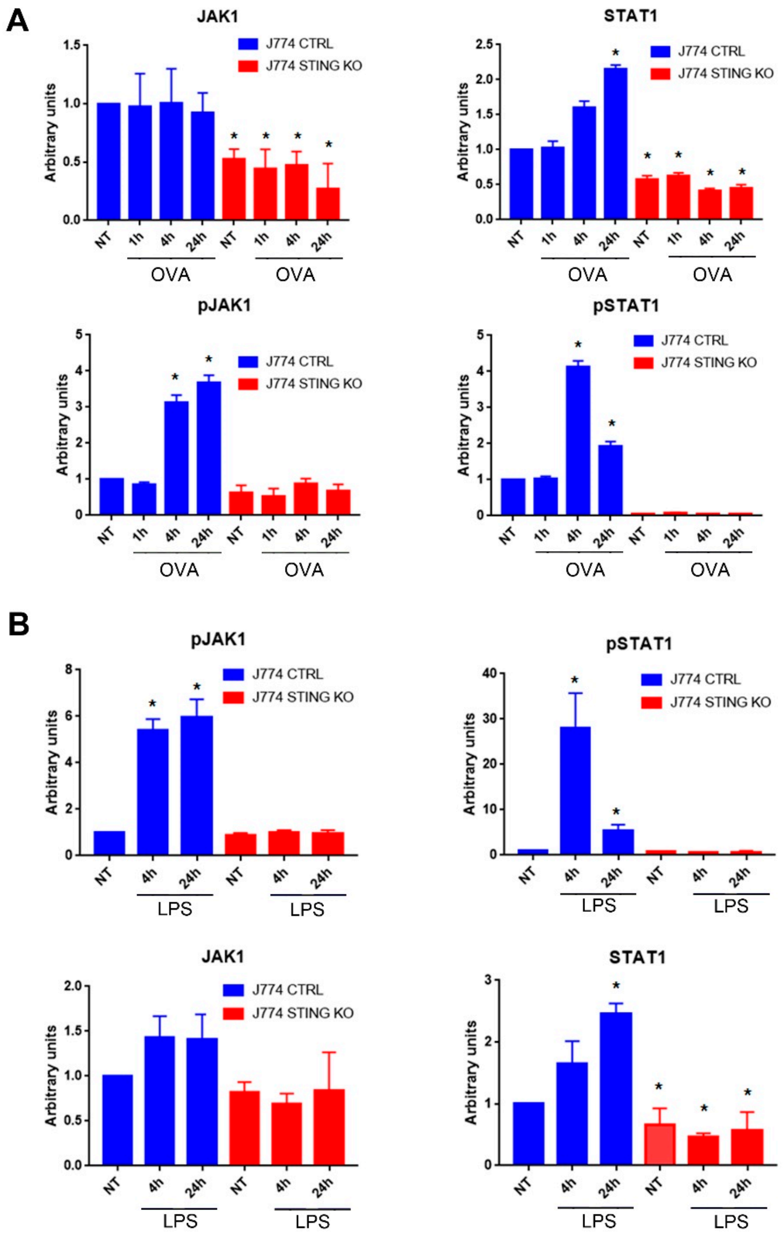

Figure S2. Quantifications of the protein levels shown in Figure 6. (A) Densitometry of the protein bands shown in Figure 6A. (B) Densitometry of the protein bands shown in Figure 6C.

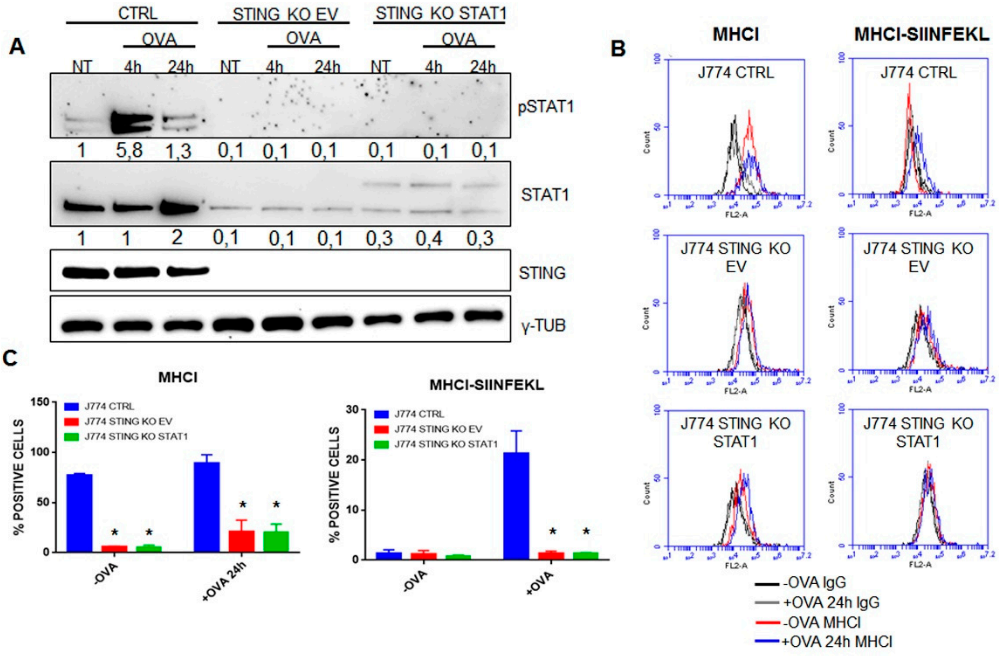

Figure S3. Overexpression of STAT1 does not rescue the MHCI levels and the antigen presentation rate in STING KO macrophages. (A) J774 CTRL, STING KO EV and STING KO STAT1 cells ($5x10^6$) were treated with 500 µg/ml of OVA or left untreated for the indicated time. Whole cell extracts (30 µg) were analyzed by western blot using the indicated antibodies. γ-Tubulin was included as control of protein loading. Mean values of the densitometry of bands are indicated. (B) J774 CTRL, STING KO EV and STAT1 ($1x10^6$) were treated with 500 µg/ml of OVA for 24h or left untreated and stained with MHC-I-PE (left panels) and SIINFEKL/H-2Kb-PE (right panel) and IgG-PE as control. Each plot represents 10000 events of a representative experiment. (C) Percentage of MHCI (left panel) and SIINFEKL/H-2Kb (right panel) positive population in untreated or OVA treated cells. Values (mean ± SE, n = 3) are shown. The asterisks indicate a statistically significant difference compared to untreated control, according to Student's *t*-test ($p \leq 0.01$).

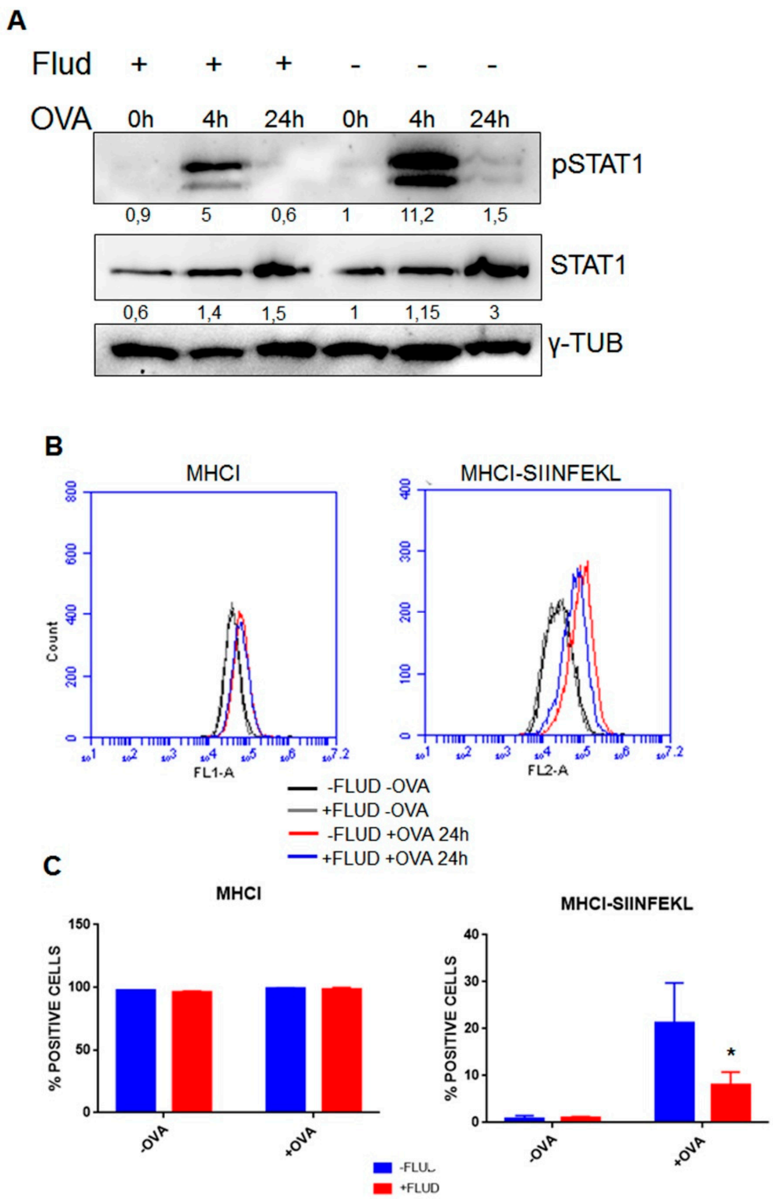

Figure S4. Fludarabine treatment reduce the rate of antigen presentation by reducing STAT1 activation. (A) J774 CTRL (5x10^6) were treated with 500 µg/ml of OVA or left untreated for the indicated time, in presence or absence of Fludarabine, as indicated. 30 µg of whole cell extracts were analyzed by western blot for the indicated proteins. γ-Tubulin was included as control of protein loading. Mean values of the densitometry of bands are indicated. (B) J774 CTRL mock and fludarabine treated (1x10^6) were incubated with 500 µg/ml of OVA for 24h and stained with MHC-I-FITC, SIINFEKL/H-2Kb-PE and IgGs (FITC and PE) as controls. Each plot represents 10000 events of a representative experiment. (C) Percentage of MHC-I (left panel) and MHC-

I-SIINFEKL (right panel) positive population in untreated or OVA treated cells in presence or absence of Fludarabine, as indicated. Values (mean ± SE, n = 3) are shown. The asterisk indicates a statistically significant difference compared to untreated control, according to Student's *t*-test ($p \leq 0.01$).
